# Patient-specific factors in the proximity of the inferior alveolar nerve to the tooth apex

**DOI:** 10.4317/medoral.18190

**Published:** 2012-08-28

**Authors:** Özkan Adigüzel, Senem Yiğit-Özer, Sadullah Kaya, Zeki Akkuş

**Affiliations:** 1 Assistant Professor, Dicle University, Faculty of Dentistry, Department of Endodontics, Diyarbakir, TURKEY; 2 Assistant Professor, Adnan Menderes University, Faculty of Dentistry, Department of Endodontics, Aydin, TURKEY; 3 Associate Professor, Dicle University, Faculty of Medicine, Department of Biostatistics, Diyarbakir, TURKEY

## Abstract

Objectives: To evaluate whether age and gender differences are predictive factors for inferior alveolar nerve position with respect to mandibular first molar roots. Study Design: Cone-beam computed tomography scans [0.2-mm3 voxel size; n = 200 (100 males, 100 females)] of patients aged 15–65 years showing mandibular first and second molars were included in this study. Patients with pathoses that might affect inferior alveolar nerve position, including second molar and/or first premolar extraction, were excluded. Fourteen measurements (mm) were taken from the inferior alveolar nerve to the mesial and distal root apices. Subjects were grouped by age and gender. Data were analysed using two-way analyses of variance with post hoc Bonferroni corrections. Results: The distance from the inferior alveolar nerve to the root apices was smaller in females than males, regardless of age (p < 0.01). Distal roots were closer to the nerve than mesial roots in both genders (p < 0.05). Total buccolingual mandibular length (at 3-mm apical level) was shorter in females than males (p < 0.01) but mean buccolingual mandibular width at the level of the inferior alveolar canal did not differ. Nerve–root apex distances were significantly shorter in males and females aged 16–25 and 56–65 years than in other age groups (p < 0.01). Conclusions: The distance between inferior alveolar nerve and mandibular first molar roots depends upon the age and gender: it is shorter in females than in males and in subjects aged 16–25 years and >55 years than in other age groups.

** Key words:**Age, cone-beam computed tomography, inferior alveolar nerve, root apex, gender.

## Introduction

The mandibular first molars are the most frequently treated teeth in endodontic procedures (1). Mental nerve paresthesia (MNP) and inferior alveolar nerve (IAN) paresthesia (IANP) due to periapical pathosis have been reported (2). The IAN, which is a branch of the mandibular nerve, is prone to injury due to trauma, tumours, and a variety of surgical treatments. Mandibular fractures, expanding compressive lesions of benign or malignant cysts, impacted teeth, local infections (e.g., osteomyelitis, peri-implant infections), anaesthetic injection, over-instrumentation or overfilling during endodontic therapy, orthognathic mandibular surgery, and dental implants have been associated with IAN injury (3,4). The proximity of the IAN to root structures is another important issue (5) that has led some practitioners to minimise the use of surgical procedures on mandibular molars (6,7).

The endodontic treatment of mandibular molars may produce localised soft-tissue paresthesia due to inadvertent apex perforation by root-canal instruments or to the use of root canal fillings and medicament systems that readily penetrate the apex when introduced into the root canal. Such iatrogenic errors may be obvious when radiographs are taken to investigate the onset of symptoms (8).

Littner et al. (9) reported that the upper border of the mandibular canal (MC) was located 3.5–5.4 mm below the root apices of the first and second molars. Levine et al. (10) showed that the IAN was 4.9 mm from the buccal cortical surface of the mandible and concluded that the bucco-lingual IAN position was associated with age and race. Denio et al. (11) found that the apex of the second molar was 3.7 mm from the upper border of the MC and that the mesial root apices of the first molars were about 6.9 mm farther from the alveolar canal. A recent study (5) that evaluated age and gender differences in the position of the IAN reported that the IAN was closer to the root apices in females than in males, regardless of age, and that gender and age were predictive factors of IAN position that should be considered in the planning of surgical procedures. According to R. Bartling et al. (12), 8,5% of IAN injury cases occur in course of dental implantation

Cone-beam computed tomography (CBCT) is an excellent preoperative diagnostic tool for visualisation of the MC (13) and the collection of accurate measurements (14-16). CBCT allows the three-dimensional reconstruction of maxillofacial structures in a fully scaled anatomical representation. The locations of the molar roots and the IAN can be evaluated in the buccolingual plane without the superimposition of surrounding anatomical structures (16). In the present study, we thus used CBCT to evaluate whether gender and age might affect the relationship of the IAN to the roots of the mandibular first molars in a Turkish popula-tion.

## Material and Methods

CBCT images (i-CAT Next Generation; Imaging Sciences International, Inc., Hatfield, PA, USA) of mandibles taken for various purposes between January 2008 and February 2011 were obtained from the radiology database of the Faculty of Dentistry, Dicle University, Diyarbakir, Turkey, and evaluated. Scans acquired using a voxel size of 0.2 mm3 were selected. Patients with extracted mandibular teeth, radiological evidence of a cyst, traumatic bone fractures, and edentulous cases were excluded. In total, 317 patients with 1502 teeth were examined. To ensure equal numbers of males and females in the sample, 200 patients with 711 teeth that met the following inclusion criteria were selected for the study: (1) known age between 15 and 65 years; (2) known gender (100 males, 100 females); (3) scan showed the left and/or right mandibular first and second molars. Patients with extracted molars were excluded.

-Transferring images to Image J

Sagittal CBCT images were saved as JPEG files and transferred to the Image J software (http://imagej.nih.gov/ij/download.html). Measurements were taken and data were saved.

-Measurements

**Figure 1:**
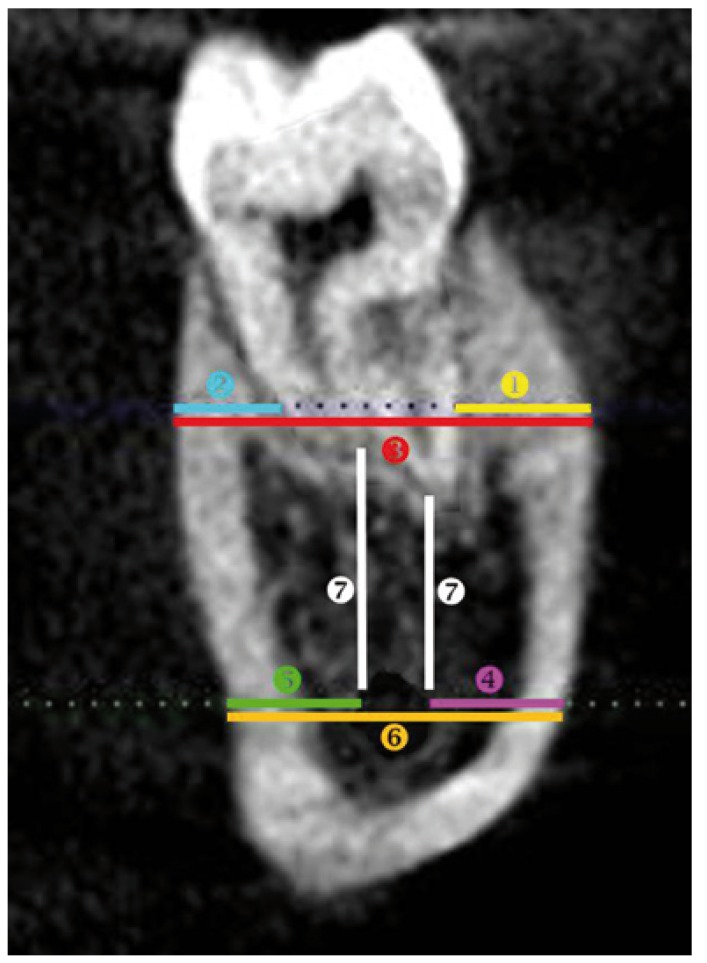
Seven measurements taken for both mesial and distal roots from the sagittal plane.

Seven anatomical measurements were taken (in mm) on each root by two observers using the sagittal planes (Fig. 1), according to the protocol described by Simonton et al. (5): (1) distance from the buccal cortical plate to the buccal aspect of the root (at 3-mm apical level), (2) distance from the lingual cortical plane to the lingual aspect of the root (at 3 mm apical level), (3) total buccolingual distance of the mandible (at 3-mm apical level), (4) distance from the buccal plate to the most buccal aspect of the inferior alveolar canal at the point perpendicular to the long axis of the root, (5) distance from the most lingual aspect of the mandibular canal to the lingual cortical plate, (6) total buccolingual width of the mandible at the level of the inferior alveolar canal, and (7) distance from the closest aspect of the root to the closest portion of the inferior alveolar canal.

To estimate intra- and interexaminer reliability, 10 patients were randomly selected and remeasured by each examiner inde-pendently with one-week interval. Then Kappa test ratios for intraexaminer and interexaminer agreements were calculated.

-Statistical analyses

Data were analysed using two-way (gender, age) analyses of variance with post hoc Bonferroni corrections. P values of less than 0.05 were considered to indicate statistical significance.

## Results

The Kappa score for interexaminer agreement after the first session was k = 0.8. After the second session per-formed 1 week later, the Kappa score for intraexaminer agreement was k = 0.9. Both scores) indicated “good agreement” ( Table 1, Table 2) present detailed results of this study, including age and gender differences. The results indicate that age and gender directly affect the planning of surgical and non-surgical endodontic treatment of the mandibular first molars.

**Table 1:**
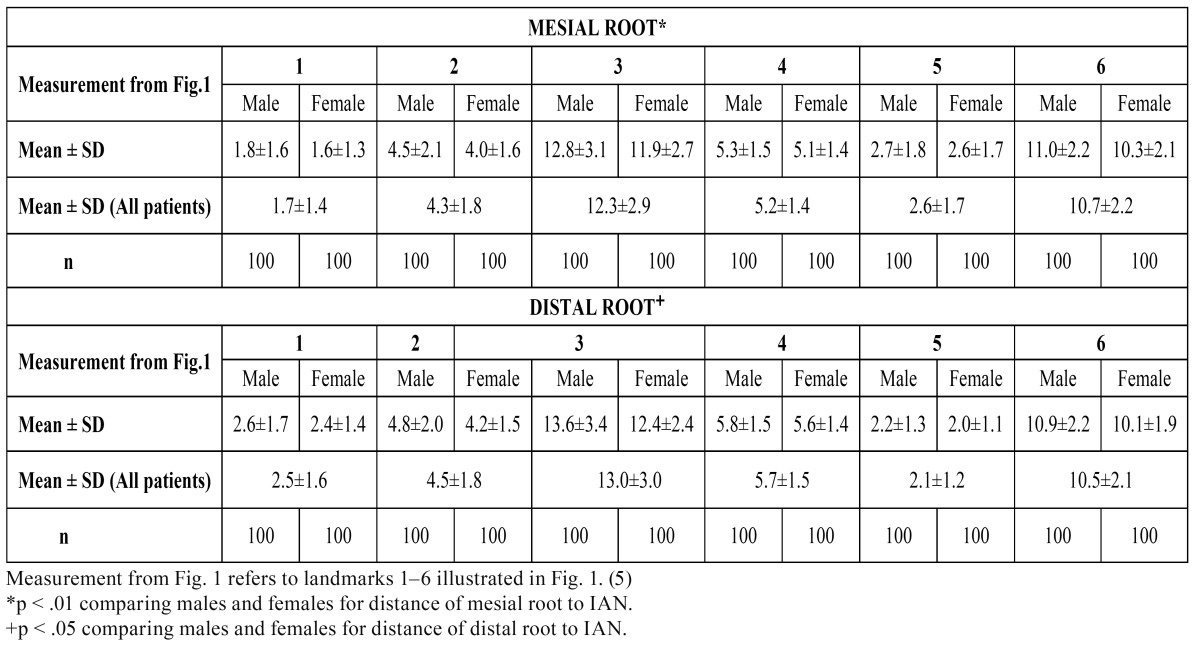
Measured Distances of Horizontal Bone Width ( mm).

**Table 2:**
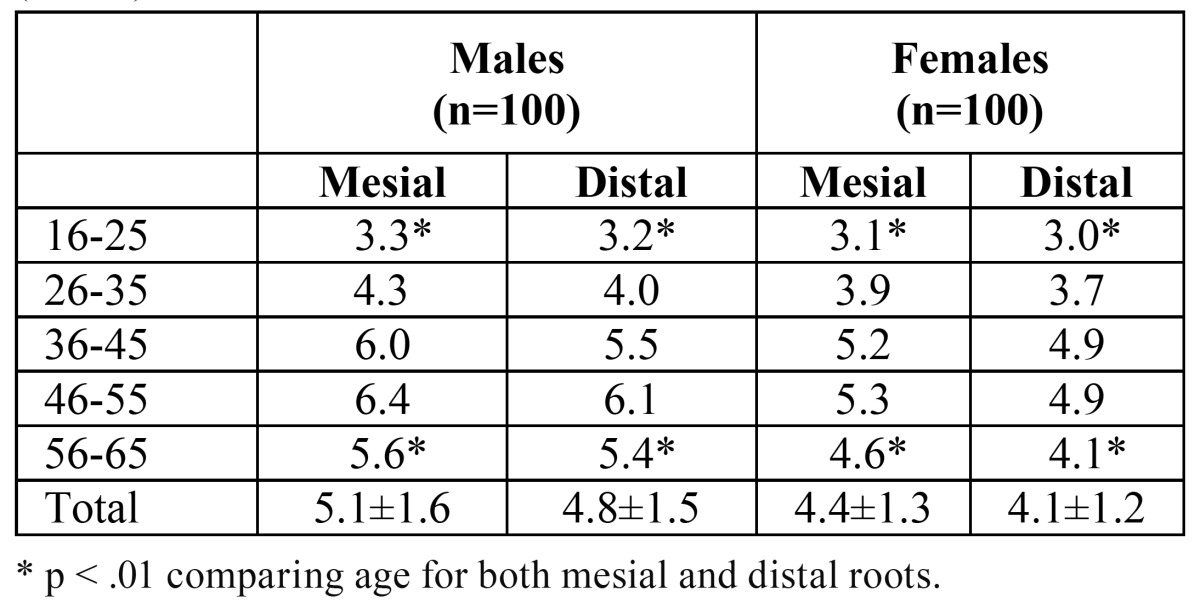
Distance of Root Apices of Mandibular First Molar to IAN (in mm).

-Gender differences

Distances from the mesial and distal root apices to the IAN were significantly shorter in females than in males, regardless of age (p < 0.01;  Table 1). The average distance from the mesial root to the IAN was 5.1±1.6 mm in males and 4.4±1.3 mm in females. The average distance from the distal root to the IAN was 4.8±1.5 mm in males and 4.1±1.2 mm in females. The distal roots were closer to the IAN in males and females (p < 0.05;  Table 1). The average total buccolingual distance of the mandible (at 3-mm apical level) was 12.3±2.9 mm for mesial roots and 13.0±3.0 mm for distal roots, and was significantly shorter in females than in males (p < 0.01). The average buccolingual width of the mandible at the level of the inferior alveolar canal was 10.7±2.2 mm for mesial roots and 10.5±2.1 mm for distal roots; this measurement did not differ significantly by gender or by root (p > 0.05).

-Age differences

**Figure 2:**
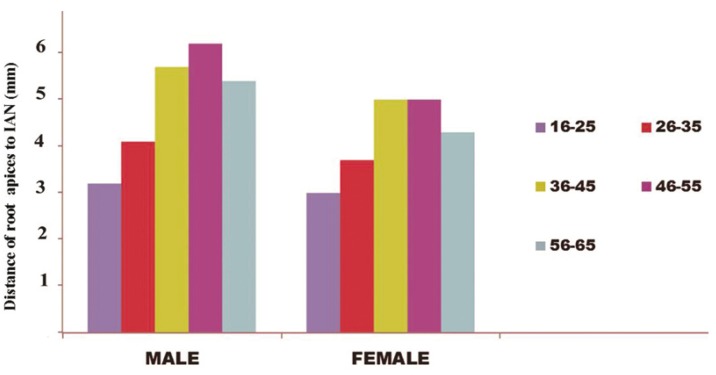
The distance of mesial and distal root apices to the closest portion of the IAN (in mm) according to age and gender.

Distances from the IAN to the root apices were significantly shorter among males and females aged 16–25 and 56–65 years than among other age groups (p < 0.01;  Table 2; Fig. 2).

## Discussion

The mandibular canal harbours a thick nerve and vessels, typically located in close proximity to the root apices (17). IAN lesions occur commonly following endodontic treatment of the mandibular molars (18,19) and may be due to root-canal instrumentation that extends beyond the apical foramen to enter the mandibular canal (20) or the forcing of root-filling material and inadequate debridement into the root and MC (21-23). The inadvertent injection of irrigation solutions, especially sodium hypochlorite, beyond the apical foramen may also cause IAN injury (3) leading to tissue necrosis (24). The extrusion of infected debris into the MC is another potential complication of root-canal treatment in mandibular molars with apical periodontitis. As reported previously (25), infected debris may breach the protective perineurium of the IAN and damage nerve conductivity. Compression arising from haematomas developing after injury to the wall or the MC contents may also result in complications, such as hypaesthesia, dysesthesia, hyperaesthesia, or complete anaesthesia (3,19). Understanding the proximity of the MC and IAN to the apices of the mandibular molar roots is thus important during and after endodontic treatments. Accurate knowledge of IAN location is essential for the prevention of iatrogenic errors.

Consistent with Simonton et al. (5), the results of the present study showed that the distances of the MC and IAN to the root apices of the mandibular first molars were shorter in females than in males. Simonton et al. (5) also reported that the distance from the IAN to the mesial root apex was shorter than that to the distal root apex, which also corresponds with the results of the present study when age-related differences are taken into account. However, these authors reported longer distances between reference points in the third through sixth decades of life, whereas our measurements indicated shorter distances. Simonton et al. (5) reported measurements of 6.2 mm for mesial roots and 5.8 mm for distal roots in males and 4.9 mm for mesial roots and 4.7 mm for distal roots in females, whereas the present study recorded measurements of 5.1 mm for mesial roots and 4.8 mm for distal roots in males and 4.4 mm for mesial roots and 4.1 mm for distal roots in females. When measuring the distance from the buccal plate to the most buccal aspect of the inferior alveolar canal at the point perpendicular to the long axis of the root Levine et al (10) reported 4.6 ± 1.1 mm for patients classified in white race and 5.9 ± 1.3 mm for non-white race. These measurements in the present study were 5.2 ± 1.4 mm for the mesial and 5.7 ± 1.5 mm for the distal roots. These differences among the studies may be due to ethnic or age differences between study populations.

Our selection of CBCT scans taken for various purposes from the radiology database resulted in the inclusion of many younger patients (16–25 years old) who had not completed skeletal growth, including 40 patients referred to orthodontic clinics. In com-parison with Simonton et al. (5), the shorter distances we observed between the IAN and the root apices of the mandibular first molars may have been due to the inclusion of such patients. Fudalej et al. (26) showed that, on average, facial growth continues in girls until about 17 years of age, whereas vertical facial growth is completed in boys at about 21 years of age. This discrepancy between males and females is related directly to the bone growth mechanism. Fudalej et al. (26) also found that the increase in anterior facial height (AFH) was about 120% greater in males than in females between the ages of 12 and 50 years. This difference may explain the shorter distances from the mesial and distal root apices to the IAN in females than in males.

The present study found that the distance from the IAN to the root apices was significantly shorter in patients of both genders aged 16–25 and 56–65 years (p < 0.01). Love et al. (27) and Foley & Mamandras (28) studied facial skeletal growth during late adolescence in males and females and reported that AFH increased over the observation period, but that the amount of increase diminished with age, especially after the age of 20 years. Between the ages of 18 and 50 years, AFH increases about 22% more in males than in females (2.2 and 1.8 mm, respectively); after the age of 50 years, vertical growth cessation rates increase (26). The findings of these investigations explain the shorter distance from the IAN to the root apices found among individuals 16–25 and 56–65 years of age in the present study.

The results of the present study were consistent with those of Denio et al. (11), who used conventional radiographic imaging to investigate the anatomical relationship of the MC with surrounding structures in mature mandibles. They found total buccolingual distances of the mandible (at 3-mm apical level) of 12.8±1.4 mm for the mesial roots and 13.6±1.2 mm for the distal roots. These distances are slightly longer than those found in the present study (12.3±2.9 mm for mesial roots, 13.0±3.0 mm for distal roots). Both studies showed that the canal wall thins anteriorly. Differences in the findings of these two studies may be due to the use of diffe-rent imaging modalities and/or ethnic variation.

## Conclusions

Distances between the IAN and the root apices of the mandibular first molars are shorter in females than in males and in individuals aged 16-25 years and >55 years than in other age groups. Age-, and gender-related variation in these distances should be appreciated by endodontists undertaking advanced surgical and non-surgical root-canal treatment of the mandibular first molars.
